# *LRRK2* and *GBA1* in Lewy body diseases: neuropathological subtypes at opposite ends of a spectrum?

**DOI:** 10.1186/s13024-026-00939-z

**Published:** 2026-04-27

**Authors:** Vedika Jha, Lorraine V. Kalia

**Affiliations:** 1 Temerty Faculty of Medicine, University of Toronto, Toronto, ON Canada; 2 Edmond J Safra Program in Parkinson’s Disease, Toronto Western Hospital, Krembil Brain Institute, University Health Network, Toronto, ON Canada

**Keywords:** Alpha-synuclein, Biomarkers, Dementia with Lewy bodies, Disease heterogeneity, GBA1, Glucocerebrosidase, LRRK2, Neuropathology, Parkinson's disease, Precision medicine

## Abstract

Lewy body diseases (LBDs), including Parkinson’s disease (PD), are defined by the presence of pathological intraneuronal α-synuclein aggregates but exhibit considerable heterogeneity in clinical course, neuropathology, and underlying mechanisms. This review summarizes neuropathological findings in PD associated with pathogenic variants in *GBA1* and *LRRK2* – the two most common genetic risk factors for PD – and highlights how these genetic forms represent neuropathological subtypes at opposite ends of a spectrum. *GBA1*-associated PD typically shows widespread Lewy pathology with cortical involvement and relatively limited Alzheimer-type co-pathology, while *LRRK2*-associated PD may occur with or without Lewy bodies and displays variability in tau and TDP-43 aggregates. We also examine how these genetic forms may serve as models for subtypes within idiopathic PD, including the potential existence of Lewy body-negative idiopathic PD. We propose a conceptual framework in which idiopathic PD encompasses *GBA1*-like and *LRRK2*-like subtypes, as well as intermediate forms with mixed pathologies. This perspective supports a shift toward biomarker-informed, mechanism-based classification of PD, beyond genetic labels alone, that may ultimately enable broader application of targeted therapeutic strategies.

**Supplementary Information:**

The online version contains supplementary material available at 10.1186/s13024-026-00939-z.

## Background

Lewy body diseases (LBDs) encompass a group of progressive neurodegenerative disorders characterized by Lewy bodies (LBs) and Lewy neurites as prominent neuropathological features. Braak staging proposes a caudal-to-rostral distribution of Lewy pathology across the nervous system. Aggregated α-synuclein is a major component of Lewy pathology, but these intraneuronal inclusions also contain a variety of other constituents, including numerous proteins, lipids and membranes, and fragmented or intact organelles such as mitochondria and components of the endolysosomal system [1–3]. These features reflect disruptions not only in proteostasis but also in broader cellular homeostasis pathways.

The two most common clinical manifestations of LBDs are Parkinson’s disease (PD) and dementia with Lewy bodies (DLB). These conditions are clinically distinguished by the temporal sequence of symptom onset, a distinction that forms the basis of current diagnostic criteria. The clinical diagnosis of PD is defined by the presence of motor parkinsonism (i.e., limb bradykinesia plus rest tremor and/or rigidity) [4]. In contrast, DLB is diagnosed when cognitive impairment occurs either before or within one year of parkinsonian motor signs, alongside core features such as fluctuating attention, visual hallucinations, and REM sleep behaviour disorder (RBD) [4, 5].

There is ongoing debate as to whether PD and DLB are distinct neurodegenerative disorders or represent a continuum of the same disease process. Arguments for separation include differences in early clinical features, biomarker profiles, and the relative timing of motor and cognitive decline [6]. However, overlapping genetic risk factors and the frequent co-occurrence of symptoms across the disease course support the view that PD and DLB may reflect different phenotypic expressions of a shared underlying pathobiology [7]. PD and DLB often exhibit converging neuropathological features upon postmortem examination, supporting the concept of a shared disease process despite clinical differences. Both typically show widespread Lewy pathology at end-stage disease, including α-synuclein aggregates in brainstem, limbic, and neocortical regions [8]. Also supporting this concept is the growing recognition that co-pathologies, such as tau, amyloid-β, and TDP-43 aggregates, frequently occur in both PD and DLB [9–13].

Most LBDs do not have a monogenic cause, but common genetic variants that significantly influence disease risk have been identified through genome-wide association studies (GWAS). For PD, GWAS have identified over 134 susceptibility loci, including *GBA1* and *LRRK2*, collectively accounting for up to approximately 28% of heritable risk, depending on the population studied [14, 15]. An additional 6 potentially novel loci have been reported in 2 recent multi-ancestry GWAS meta-analyses [16, 17]. GWAS investigating DLB have identified five independent risk loci, each implicated in Alzheimer’s disease (AD) or PD, including *APOE*, *BIN1*, *TMEM175*, *SNCA*, and *GBA1* [18]. 

**Fig. 1:**
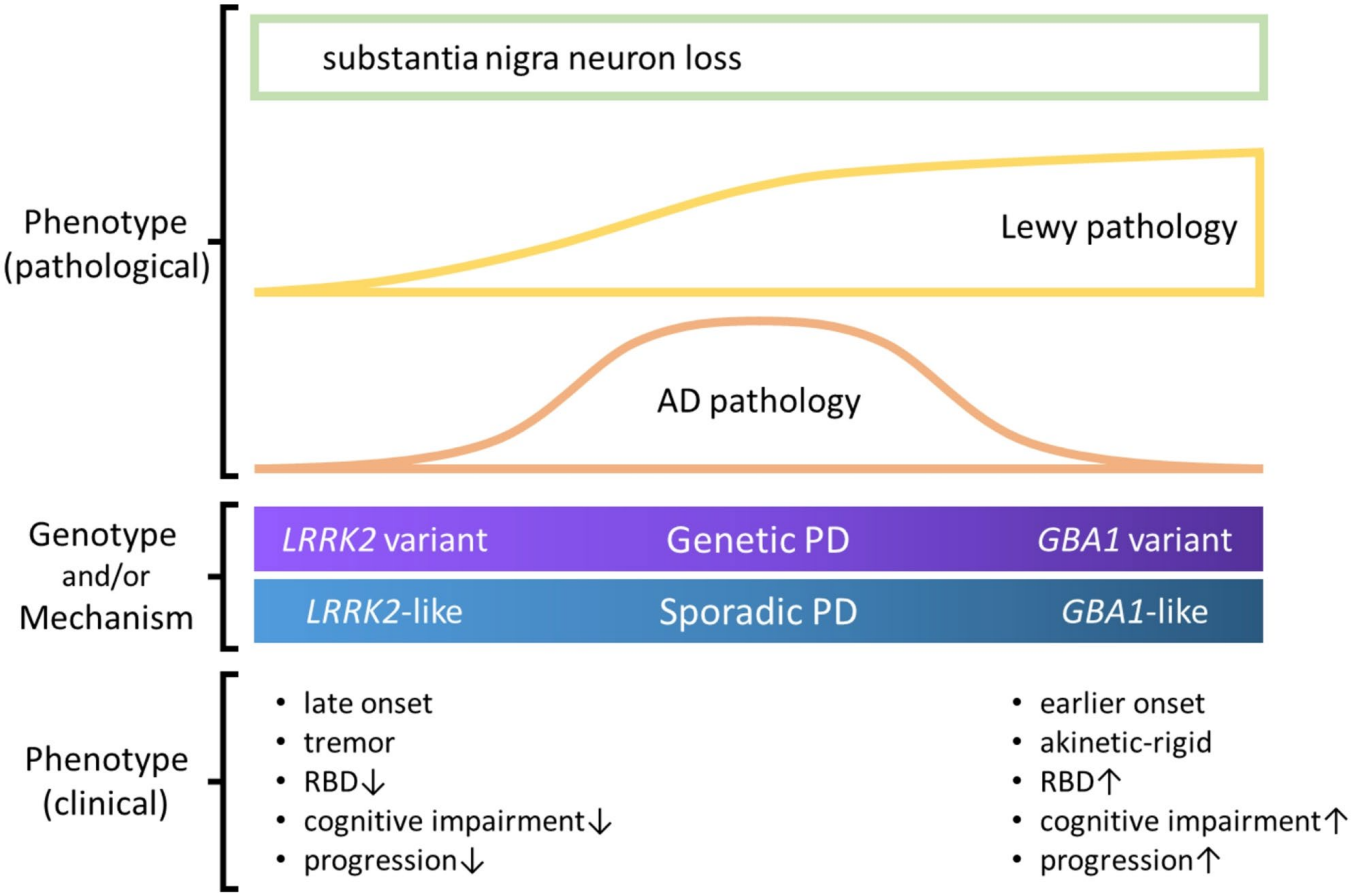
A proposed spectrum for Lewy Body Diseases based on *LRRK2*- and *GBA1*-associated Parkinson’s disease. *LRRK2-* and *GBA1*-associated PD define opposite neuropathological extremes (LB-negative to LB-abundant, with prominent substantia nigra neurodegeneration but low AD co-pathology in both), mirrored by distinct clinical phenotypes. This spectrum extends to idiopathic or sporadic PD, with “*LRRK2*-like” and “*GBA1*-like” subtypes and increasing co-pathology in the intermediate range

*GBA1* encodes the lysosomal enzyme glucocerebrosidase (GCase) [15, 19, 20]. Experimental studies suggest that reduced enzymatic activity due to disease-associated variants promotes α-synuclein accumulation and aggregation. *GBA1* variants are found in approximately 7% to 12% of PD patients in European ancestry cohorts [19, 20]. *GBA1*-associated PD tends to be characterized by earlier disease onset, akinetic-rigid motor features, higher burden of non-motor features (e.g., RBD, cognitive impairment), and more rapid progression compared with idiopathic PD, suggestive of more diffuse Lewy pathology [21] (Fig. 1**)**.

*LRRK2* encodes leucine-rich repeat kinase 2, a large multidomain protein with both kinase and GTPase activity that regulates vesicular trafficking, autophagy, and intracellular signaling. Pathogenic variants, particularly the p.G2019S substitution, are present in approximately 1% to 2% of PD patients of European ancestry [19, 20] and are enriched in certain founder populations such as Ashkenazi Jews and North African Arabs. The clinical features of *LRRK2*-associated PD are generally distinguished by a relatively tremor-predominant motor phenotype, slower progression, and lower burden of non-motor features, including less frequent RBD and cognitive impairment, compared with idiopathic PD [22] (Fig. 1). Consistent with this clinical profile, *LRRK2* variants have not been found to be causal for DLB [23, 24]. Notably, *LRRK2* carriers exhibit diverse neuropathological findings, ranging from classical Lewy pathology to nigral degeneration in the absence of LBs [25].

The goal of this review is to synthesize neuropathological findings from individuals with *GBA1*- and *LRRK2*-associated LBD and to explore how these genetic forms may represent neuropathological subtypes at opposite ends of a spectrum. We propose that *GBA1*-associated LBD is characterized by widespread α-synuclein pathology and frequent cortical involvement, resembling a DLB-like phenotype, while *LRRK2*-associated LBD may in some cases represent a LB-negative disease. Furthermore, we speculate that these forms may model underlying subtypes within idiopathic PD, offering a framework for identifying “*GBA1*-like” and “*LRRK2*-like” cases based on clinical and biomarker features. This perspective, based on neuropathological subtypes, has important implications for advancing our understanding of disease mechanisms, heterogeneity, and progression across the LBD spectrum, as well as for targeted therapeutic development.

## *GBA1*-associated Lewy body disease

The *GBA1* gene encodes the lysosomal enzyme GCase, which cleaves glycosphingolipids into glucose and ceramide [26]. Biallelic pathogenic variants in *GBA1* cause Gaucher disease (GD), an autosomal recessive lysosomal storage disorder characterized by the accumulation of glycosphingolipids within lysosomes across multiple cell types [27]. GD exhibits a broad spectrum of clinical severity and may remain minimally symptomatic or asymptomatic in some individuals. It is typically classified into three subtypes: type 1 (non-neuronopathic) is the most common form and was previously considered to lack central nervous system (CNS) involvement, while type 2 (acute neuronopathic) and type 3 (chronic neuronopathic) are more severe, present early in life, and are defined by prominent CNS manifestations [28]. The association between *GBA1* variants and LBDs was established through multicentre studies demonstrating an increased prevalence of PD among individuals with type 1 GD [29, 30]. It is now also recognized that heterozygous *GBA1* carriers, who do not develop GD, have a higher prevalence of both PD and DLB compared to non-carriers [31]. Several mechanisms have been proposed to explain how *GBA1* variants contribute to LBD pathogenesis, including both loss-of-function and gain-of-function hypotheses [32, 33]. However, the precise role of *GBA1* in LB formation and in the pathophysiology of PD and DLB remains incompletely understood. Nevertheless, postmortem studies of brains from individuals with *GBA1*-associated PD suggest a close association between *GBA1* variants and α-synuclein-related pathology.

### Neuropathological findings in *GBA1*-associated Lewy body disease

Neuropathological data from at least 19 studies encompassing 310 individuals – 280 *GBA1* variant carriers and 30 GD patients – have been reported to date (Table 1). Across these studies, 97% (*n* = 300) of individuals exhibited some form of α-synuclein-related pathology. Among them, 78 (25%) had a neuropathological diagnosis of PD or PD dementia (PDD), and 38 individuals (9%) were diagnosed with DLB. An additional 174 individuals (56%) were diagnosed with LBD without specification of PD versus DLB. When Braak staging was available, 84% of cases (107 out of 127) were classified as stage four to six, indicating cortical involvement (Table 2). Not all studies included clinical diagnostic information. For example, the largest cohort (*n* = 165) reported by Walton and colleagues consisted of pathologically confirmed LBD cases, but specific clinical diagnoses were not available [34].

**Table 1:** Summary of *GBA1* autopsy reports (adapted from Schneider et al. 2017)

Report	*N*	Genotype (*n*)	Clinical diagnosis (*n*)	Pathological diagnosis (*n*)	Lewy pathology (*n*)	Braak stage (*n*)	Tau pathology/ NFT stage (*n*)	Other results
Li et al. 2019	9	Heterozygote (9)	No details.	PD (9)	Present (9)	Mean 5.5 *±* 0.8	No details.	Accumulation of oligomeric α-synuclein on Western blot in both *GBA1* PD and non-*GBA1* PD. Mitophagy defects in *GBA1* PD.
Monestime et al. 2016	5	Homozygote (1) Compound heterozygote (4)	GD + PD (3) GD + DLB (2)	PD (2) DLB (3)	Present (3)	No details.	No details.	High degree of clinical variability. All treated with enzyme replacement therapy.
Kurzawa-Akanbi et al. 2021	7	Heterozygote (7)	No details.	PD (4) PDD (3)	Present (7)	4 (2) 2 (2) 0 (2) Not available (1)	No details.	No details.
Moors et al. 2019, Moors et al. 2024	10	Heterozygote (9) Unknown (1)	PD (6) DLB (4)	PD (6) DLB (4)	Present (10)	6 (4) 5 (4) 4 (2)	I (10)	Decreased cathepsin D activity in *GBA1* PD versus non-*GBA1* PD. Frequent transcription factor EB clusters at the Golgi apparatus in *GBA1* and non-*GBA1* PD compared to controls.
Tayebi et al. 2023, Wong et al. 2004, Goker-Alpan et al. 2010	4	Homozygote (2) Compound heterozygote (1) Unknown (1)	GD + PDD (4)	PDD (4)	Present (4)	5 to 6 (2) 3 (1) 4 (1)	No details.	GCase present in a similar pattern to LBs and LNs. GCase largely in LB core in brainstem versus co-localized with α-synuclein in cortical LBs.
Lwin et al. 2004	12	Homozygote (2)	GD + PD (2)	No details.	Present (2)	6 (2)	No details.	GCase enzyme activity 7% to 11% in GD and 43% to 100% in *GBA1* carriers.
		Heterozygote (10)	PD (10)	No details.	Present (8)	6 (5) 4 (3) 0 (2)	No details.	No details.
Eblan et al. 2005	2	Heterozygote (2)	PD (2)	PD (2)	No details.	No details.	No details.	No details.
Goker-Alpan et al. 2006, Goker-Alpan et al. 2010	9	Heterozygote (9)	PDD (9)	DLB (3) DLB + AD (6)	Present (9)	6 (8) 4 (1)	AD (6)	See above.
Mata et al. 2008	2	Heterozygote (2)	No details.	PDD (2)	Present (2)	6 (2)	II (1) V (1)	No details.
Clark et al. 2009	34	Homozygote (1)	GD (1)	AD (1)	Absent (1)	0 (1)	*≥*III	No details.
		Heterozygote (25) Homozygote (1) Unknown (1)	No details.	DLB (22) LBVAD (5)	Present (27)	4 to 6 (26) 3 (1)	*≥*III (5) <II (22)	No details.
		Heterozygote (5)	No details.	AD (5)	Absent (5)	0 (5)	*≥*III	No details.
		Heterozygote (1)	No details.	Normal (1)	Absent (1)	0 (1)	No details.	No details.
Farrer et al. 2009	8	Heterozygote (8)	PD (2) DLB (6)	LBD (8)	Present (8)	5 to 6 (6) 4 (2)	No details.	No details.
Segarane et al. 2009	1	Heterozygote (1)	MSA (1)	MSA (1)	No details.	No details.	No details.	No details.
Neumann et al. 2009, Gegg et al. 2012, Gegg et al. 2015	17	Heterozygote (17)	PD (15) MSA (2)	PD (17)	Present (17)	5 (4) 6 (13)	>III (2) <II (15)	No total changes in ceremide levels between controls, *GBA1* PD, or non-*GBA1* PD. Greatest decrease in GCase activity in SN.
Nishioka et al. 2011	4	Heterozygote (3) Homozygote (1)	PDD (4)	LBD (4)	Present (4)	5 to 6 (4)	No details.	No details.
Sklerov et al. 2017	1	Homozygote (1)	MSA (1)	MSA (1)	Absent (1)	0 (1)	No details.	Diffuse glial cytoplasmic inclusions. Occasional neurons labeled with anti-α-synuclein antibodies without LBs.
	3	Heterozygote (3)	MSA (3)	MSA (3)	Absent (3)	0 (3)	Rare neurofibrillary tangles (2) Rare tau positive neurons (2)	
Blauwendraat et al. 2019	1	Homozygote (1)	PD (1)	AD (1)	Absent (1)	0 (1)	AD, intermediate level (1)	Neuronal loss in SN.
	4	Heterozygote (4)	PDD (2) DLB (1) PD + AD (1)	PD (1) LBD + AD (3)	Present (4)	5 to 6 (4)	AD, low level (3) AD, intermediate level (1)	No details.
Adler et al. 2017	12	Homozygote (1) Compound heterozygote (1) Heterozygote (10)	PD (2) PDD (3) PDD + AD (7)	PD (2) PDD (3) PDD + AD (7)	Present (12)	6 (12)	No details.	No details.
Walton et al. 2017	165	Homozygote or compound heterozygote (16) Heterozygote (149)	No details.	LBD (165)	Present (165)	No details.	No details.	*GBA1* variants, especially common variants, such as p.N409S and p.L483P, were associated with decreased tau pathology/NFT stage but increased likelihood of dementia (associations, not raw numbers, were reported).

AD=Alzheimer’s Disease, DLB=Dementia with Lewy Bodies, GCase = Glucocerebrosidase, GD=Gaucher’s Disease, LB= Lewy Body, LBD=Lewy Body Disease, LBVAD=Lewy Body Variant of Alzheimer’s Disease, LN=Lewy Neurite, MSA=Multiple System Atrophy, NFT= Neurofibrillary Tangle, PD=Parkinson’s Disease, PDD=Parkinson’s Disease Dementia, SN = Substantia Nigra

**Table 2:** Braak stages of Lewy body pathology for Gaucher disease patients and *GBA1* carriers

Braak stage	Distribution of Lewy pathology	Gaucher disease	*GBA1* carriers	*N*
0	No Lewy pathology	3	13	16
1	Dorsal motor nucleus of the vagal nerve	0	0	0
2	Raphe nuclei, locus ceruleus	0	2	2
3	Midbrain, substantia nigra	1	1	2
4	Prosencephalon, cortical involvement confined to temporal mesocortex	1	10	11
4 to 6	Cortical involvement	0	26	26
5	High order sensory association areas of the neocortex and prefrontal neocortex	0	8	8
5 to 6	Neocortical involvement	2	14	16
6	First order sensory association areas of the neocortex and premotor areas	2	44	46
Not reported		21	162	183
Totals		30	280	310

Braak staging reflects the anatomical distribution of Lewy pathology, ranging from stage 0 (no pathology) to stage 6 (extensive neocortical involvement). Cases with unreported Braak stage are included in the “Not reported” category

Other reported neuropathological diagnoses included AD and multiple system atrophy (MSA). Five cases (2%) were diagnosed with MSA, none of which exhibited concomitant Lewy pathology. These cases may represent rare *GBA1*-associated MSA or genetic non-penetrance of *GBA1* variants for Lewy body disease. Most of these studies were not specifically designed to assess the relationship between *GBA1* variants and MSA, but an analysis of 108 neuropathologically confirmed MSA cases by Segarane and colleagues identified only one individual (0.9%) with a heterozygous *GBA1* variant, compared to three individuals (1.2%) in the control group, supporting a lack of association between *GBA1* status and MSA [35]. Co-pathology with AD was uncommon, although under-reporting cannot be excluded. Nine cases with a neuropathological diagnosis of PDD, DLB, or LBD also met criteria for AD. In addition, five cases were classified as LB variant of AD, and seven were diagnosed with AD in the absence of LB pathology. Tau pathology was documented for 77 cases, but only 21 of them (27%) had sufficient neurofibrillary tangle (NFT) burden to meet pathological diagnostic criteria for AD. These findings suggest that while tau pathology may be present in *GBA1*-associated disease, the overall burden is typically low. This is consistent with observations from Walton and colleagues who found a negative association between *GBA1* variants and NFT burden [34].

### Comparison between Gaucher disease and *GBA1* carriers

Few consistent neuropathological differences have been identified between individuals with GD and heterozygous *GBA1* variant carriers. Among the 30 reported cases with homozygous or compound heterozygous *GBA1* variants, 26 (87%) exhibited LB pathology. Notably, two GD patients with homozygous p.N409S variants had a clinical diagnosis of AD or MSA, rather than PD, and did not show Lewy pathology [36, 37]. Among the 280 *GBA1* carrier cases, 272 (97%) exhibited LB pathology. Of the remaining cases, two were diagnosed with PD but lacked LBs, while the others included five AD cases, four MSA cases, and one case without pathological features of a neurodegenerative disease.

Only one study has examined the neuropathological features of LBD across GD subtypes. Wong and colleagues assessed four individuals with both PD and either type 1, 2, or 3 GD and found common pathological features, including expected neuronal loss and brainstem-type LBs in the substantia nigra (SN), along with less typical gliosis in the hippocampal CA2-4 regions [38]. Some subtype-specific patterns were observed: patients with type 1 GD had prominent astrogliosis without marked neuronal loss, whereas those with type 2 or 3 exhibited both astrogliosis and substantial neurodegeneration [38].

### *GBA1* variant-specific neuropathological findings

Among the postmortem studies of brains from GD patients and heterozygous *GBA1* carriers, a total of 44 distinct *GBA1* genotypes were reported (Table 3). Walton and colleagues identified an additional 27 unique variants in their large autopsy series, presented with allele frequencies in Supplementary Table 1 [34]. Of the 64 unique variants, 36 (56%) had been previously reported and were catalogued in the gnomAD database. Many of these variants are well characterized in terms of their association with GD, increased risk of PD, or both, although risk estimates vary across studies [32]. The most frequently reported variants were p.N409S, p.E365K, p.T408M, and p.L483P. Notably, some variants increase PD risk but are not associated with GD (e.g., p.E365K, p.T408M). p.N409S was the most prevalent overall, with 32 heterozygotes, six homozygotes, and four compound heterozygotes identified. Among the 14 GD patients with available data, 12 (86%) carried at least one N409S allele with 9 (64%) being homozygous for this variant.

**Table 3:** Summary of *GBA1* genotypes reported in autopsy studies and their variant classification

Genotype	*N*	Variant type	Consequence	ClinVar classification
p.N409S/wt	32	Heterozygous	Missense	Pathogenic/Likely pathogenic & Risk factor
p.E365K/wt	20	Heterozygous	Missense	Conflicting classifications of pathogenicity & risk factor
p.T408M/wt	16	Heterozygous	Missense	Conflicting classifications of pathogenicity
p.L483P/wt	14	Heterozygous	Missense	Pathogenic/Likely pathogenic
p.N409S/p.N409S	9	Homozygous	Missense	Pathogenic/Likely pathogenic & Risk factor
p.D448H/wt	5	Heterozygous	Missense	Pathogenic/Likely pathogenic
p.R502C/wt	4	Heterozygous	Missense	Pathogenic
p.IVS2 + 1/wt	3	Heterozygous	Splice site	Pathogenic/Likely pathogenic
p.N409S/c.84insG	2	Compound heterozygous	Frameshift, termination	Pathogenic/Likely pathogenic & Risk factor; Absent
p.D448H/p.L483P	2	Compound heterozygous	Missense	Pathogenic/Likely pathogenic
p.R301H/wt	2	Heterozygous	Missense	Uncertain significance
RecNciI/wt	2	Heterozygous	Recombination	Pathogenic/Likely pathogenic & Risk factor
p.H294Q/wt	2	Heterozygous	Missense	Conflicting classifications of pathogenicity
p.R532H/wt	2	Heterozygous	Missense	Absent
p.E427K/wt	2	Heterozygous	Missense	Uncertain significance
c.762-18T > A /wt	1	Heterozygous	Intronic	Absent
p.N409S/p.L483P	1	Compound heterozygous	Missense	Pathogenic/Likely pathogenic & Risk factor
p.L144R/wt	1	Heterozygous	Missense	Absent
p.P358L/wt	1	Heterozygous	Missense	Absent
p.D179H/wt	1	Heterozygous	Missense	Uncertain significance
p.N409S/?	1	Unknown	Missense	Pathogenic/Likely pathogenic & Risk factor
p.K237T/wt	1	Heterozygous	Missense	Absent
p.R368C/wt	1	Heterozygous	Missense	Conflicting classifications of pathogenicity
p.T306I + p.E365K/wt	1	Heterozygous	Missense	Conflicting classifications of pathogenicity & Risk factor; Uncertain significance
p.I200N/wt	1	Heterozygous	Missense	Absent
p.A398X/wt	1	Heterozygous	Stop gain	Pathogenic
p.R159W/wt	1	Heterozygous	Missense	Absent
c.84insG/wt	1	Heterozygous	Frameshift, termination	Absent
p.W223R/wt	1	Heterozygous	Missense	Pathogenic/Likely pathogenic
p.E365K + p.N217R + p.S225P + p.V220G	1	Unknown	Missense	Conflicting classifications of pathogenicity & Risk factor; Absent; Pathogenic/Likely pathogenic; Unknown
p.T408M/p.T408M	1	Homozygous	Missense	Conflicting classifications of pathogenicity
p.G1444 A > G/wt	1	Heterozygous	Non-coding substitution	Absent
p.P220P/wt	1	Heterozygous	Synonymous	Absent
p.G428V/wt	1	Heterozygous	Missense	Absent
c.1263–1317del/wt	1	Heterozygous	Non-coding deletion	Absent
p.R170C/wt	1	Heterozygous	Recombination	Pathogenic
p.C232E/wt	1	Heterozygous	Missense	Pathogenic
RecA456P/wt	1	Heterozygous	Recombination	Absent
p.A331T/p.A331T	1	Homozygous	Missense	Absent
p.T362I/wt	1	Heterozygous	Missense	Pathogenic/Likely pathogenic
p.R87Q/wt	1	Heterozygous	Missense	Uncertain significance
p.S235P/wt	1	Heterozygous	Missense	Pathogenic/Likely pathogenic
p.T408M/p.V486E	1	Compound heterozygous	Missense	Conflicting classifications of pathogenicity; Absent

Pathogenicity and risk factor status are reported according to current ClinVar entries, where available. Data from Walton et al. 2024 are excluded due to a lack of genotype reporting but allele frequencies are presented in Supplementary Table 1

Walton and colleagues evaluated associations between common *GBA1* variants and specific clinical and neuropathological outcomes [34]. p.E365K was significantly associated with increased odds of dementia (OR = 1.88, 95% CI [1.08, 3.26]). Other common variants, including p.T408M, p.N409S, p.L483P, and c.762-18T > A, also showed elevated odds for dementia, though these did not reach statistical significance. A gene-burden analysis of rare *GBA1* variants (minor allele frequency < 0.5%) revealed a significant association with dementia. Interestingly, some *GBA1* variants appeared to confer protection against AD pathology. They reported that both p.N409S and p.L483P were associated with significantly lower NFT burden, with odds ratios of 0.23 (95% CI [0.08, 0.65]) and 0.10 (95% CI [0.03, 0.35]), respectively [34]. These findings support the broader hypothesis that *GBA1* variants preferentially increase vulnerability to α-synuclein-related pathology over tau-mediated neurodegeneration.

Several clinical studies have classified p.N409S and p.E365K as mild variants based on their relatively lower odds ratios for PD, while p.L483P is considered a severe variant with stronger associations to PD. Moreover, one study found that carriers of severe variants, such p.L483P, had a significantly elevated risk of developing dementia [39]. However, because the study by Walton and colleagues did not include corresponding clinical data, the extent to which these variant-specific neuropathological findings correlate with clinical outcomes remains uncertain.

### Neuropathological differences between *GBA1*-associated and idiopathic Parkinson’s disease

Studies have compared postmortem brain tissue from individuals with *GBA1*-associated PD to those with idiopathic PD. Some reported a higher prevalence or greater density of cortical LBs in *GBA1* variant carriers compared to non-carriers [34, 36], while others found no significant difference [37, 39–41]. These discrepancies may reflect limitations such as small sample sizes, variability in neuropathological assessment, and differing analytic methods. As noted above, the large autopsy cohort reported by Walton and colleagues found that *GBA1* variants were associated not only with increased odds of dementia but also with less severe AD pathology, suggesting that *GBA1* variants preferentially contribute to synucleinopathy rather than tauopathy [34].

Differences in LB composition have also been observed. One study used immunohistochemistry to examine GCase localization within LBs and found that 75% of LBs in *GBA1* heterozygotes and over 80% in homozygotes contained GCase, compared to fewer than 10% of LBs in idiopathic PD cases [42]. In *GBA1-*associated cases, GCase was localized to the core of brainstem LBs and co-localized with α-synuclein in cortical LBs, with this pattern more prominent in homozygous individuals [42].

While *GBA1* variants may directly impair GCase function, several lines of evidence from studies examining idiopathic PD brain tissue suggest that similar lysosomal and lipid metabolic dysfunction occurs even in the absence of *GBA1* variants. Abnormal glycosphingolipid accumulation has been reported in idiopathic PD brains, although direct comparisons between idiopathic and *GBA1*-associated PD have not consistently revealed differences in lipid profiles [43]. Enzymatic studies have shown reduced GCase activity and Golgi dysfunction in the SN of both idiopathic and *GBA1*-associated PD brains [44]. Additional work has identified lower GCase protein levels in the SN and cerebellum in both groups, with further reductions in the putamen observed only in *GBA1*-associated PD [45]. One hypothesis that has been proposed from these findings is that mitochondrial dysfunction and oxidative stress may underlie the reduction in GCase activity in idiopathic PD, and that this lysosomal stress pathway may be further exacerbated in *GBA1*-associated PD. Supporting this, mitophagy defects have been documented in both idiopathic and *GBA1*-associated PD, with the latter showing higher levels of 8-OHdG, a marker of oxidative DNA damage, and more pronounced mitochondrial abnormalities [46]. Together, these findings suggest that *GBA1*-associated PD may represent a more severe or accelerated form of lysosomal-mitochondrial dysfunction observed in idiopathic PD.

## *LRRK2*-associated Parkinson’s disease

The *LRRK2* gene encodes leucine-rich repeat kinase 2 (LRRK2), a large multidomain protein in the ROCO family that is defined by the presence of ROC (Ras of complex proteins) and COR (C-terminal of ROC) domains and exhibits both GTPase and serine/threonine kinase activities [47]. Pathogenic *LRRK2* variants are associated with an autosomal dominant form of PD with most mutations affecting its catalytic core, leading to dysregulated enzymatic activity and downstream signaling alterations. The most common pathogenic variants include p.R1441C/G/H and p.Y1699C (within the ROC-COR tandem domains), as well as p.G2019S and p.I2020T (within the kinase domain) [47]. While *LRRK2* variants are well-established genetic contributors to PD, they have not been implicated as causal in DLB through linkage or segregation studies, nor have they been shown to increase its risk in GWAS [23], although the statistical power of existing studies to detect modest effects in DLB is limited. In contrast, recent findings from European cohorts suggest that an intronic variant tagging *LRRK2* p.G2019S may in fact confer protection against DLB [24], consistent with earlier research demonstrating that *LRRK2* p.G2019S carriers are less likely to develop dementia [48]. LRRK2 influences a broad range of cellular processes, including the endolysosomal system (particularly through the phosphorylation of Rab GTPases and regulation of lysosome size, number, and degradative capacity), cytoskeletal dynamics, and mitochondrial quality control. Its expression patterns within the CNS vary significantly by cell type, being prominently expressed in non-neuronal cells like microglia, and in excitatory neurons and oligodendrocyte precursors in humans, rather than primarily in dopaminergic neurons themselves [47]. Experimental models have shown that *LRRK2*-mediated neurodegeneration can occur either independently of, or in conjunction with, α-synuclein aggregation [49]. This underlying complexity is reflected in the neuropathological heterogeneity observed in *LRRK2*-associated PD, particularly regarding the presence of classical α-synuclein-positive Lewy pathology [25].

### Neuropathological spectrum in *LRRK2*-associated Parkinson’s disease

The largest clinicopathological correlation study of *LRRK2*-associated PD to date evaluated 37 cases selected from an initial cohort of 59 autopsied individuals. Twenty-two cases were excluded due to the presence of non-pathogenic variants, non-manifesting carrier status, or insufficient clinical and/or pathological data [25]. Among the 37 included cases, 17 (46%) exhibited Lewy pathology, whereas 20 (54%) did not. Within the p.G2019S subgroup, 11 cases (65%) were LB-positive, while 6 (35%) were LB-negative. Notably, other pathogenic *LRRK2* variants demonstrated a greater propensity for LB-negative pathology: 8 of 9 p.I2020T cases (89%) and 4 of 6 p.R1441C/G cases (67%) lacked LBs. Clinicopathological correlations revealed that tremor was the most common presenting symptom for PD patients with pathogenic *LRRK2* variants regardless of LB pathology (65% for both LB-positive and LB-negative groups). Although these findings suggest that α-synuclein-negative pathology occurs in a notable subset of *LRRK2*-associated PD cases, the reported proportions likely overstate its true prevalence due to ascertainment and publication bias, and therefore should be interpreted with caution. Despite this heterogeneity, degeneration of the SN was consistently observed across all cases, regardless of LB status. Cardinal motor symptoms, atypical features, levodopa responsiveness, and motor complications occurred at similar frequencies across LB-positive and LB-negative groups. Certain non-motor features were more commonly observed in LB-positive cases. After adjusting for disease duration and age at death, the presence of cognitive impairment or dementia, anxiety, and orthostatic hypotension remained significantly associated with Lewy pathology, even after accounting for Alzheimer-type pathology [25]. Together, these findings reinforce that LBs are neither necessary nor sufficient for the motor symptoms of *LRRK2*-associated PD but may contribute to cortical dysfunction underlying specific non-motor features, such as cognitive decline.

Beyond the variability in α-synuclein pathology, *LRRK2*-associated PD exhibits additional heterogeneity with respect to tau and TDP-43 co-pathologies. A recent report of 12 individuals from the Sagamihara family carrying the *LRRK2* p.I2020T mutation found that pure nigral degeneration without LBs was the most common neuropathological finding, observed in 8 of 12 cases (67%) [50]. Among the four cases with α-synuclein pathology, one fulfilled a neuropathological diagnosis of MSA. Tau pathology in these p.I2020T cases was limited, typically restricted to a few brainstem NFTs consistent with age-related change. Phosphorylated TDP-43 aggregates were identified in 5 of 12 cases (42%), a frequency not significantly higher than that reported in healthy older adults or typical LBD. The spatial pattern of these aggregates, localized to the SN but not limbic regions, differed from both age-related TDP-43 proteinopathy and LBD, leading the authors to propose that the TDP-43 pathology in this family could be a secondary change [50]. A separate study found non-phosphorylated TDP-43 aggregates in 73% of examined *LRRK2* p.G2019S brains (8 of 11), compared to only 18% in idiopathic PD brains [51]. Tau pathology has been variably reported in *LRRK2* p.G2019S cases, but no systematic study has compared its prevalence or burden to that seen in idiopathic PD, raising the possibility of overestimation. There have been a few reports of cases with tufted astrocytes similar to PSP, and *LRRK2* variants have been detected in pathologically confirmed primary tauopathies, albeit with very low frequency [52]. Collectively, these findings suggest that while tau and TDP-43 pathologies may co-occur in *LRRK2*-associated PD, they are unlikely to represent major contributors to disease.

### Lewy body-negative Parkinson’s disease

Based on the studies described above and others, it is now well-established that Lewy pathology is absent in a significant subset of individuals with *LRRK2*-associated PD which raises several important questions regarding the nature of α-synuclein pathology and its relevance to disease mechanisms. First, does the absence of LBs at autopsy reflect a true lack of pathological α-synuclein in vivo over the course of the disease? Second, if α-synuclein does contribute to neurodegeneration in LB-negative cases, in what form does it exist, and how might it mediate neuronal loss? Third, could the sequestration of α-synuclein into LBs represent an epiphenomenon rather than a central mediator of neurodegeneration, thereby accounting for the variable presence of Lewy pathology across clinical and genetic forms of PD? Finally, this pathological heterogeneity raises a broader and still unresolved question: can idiopathic PD lacking Lewy pathology represent a distinct pathological entity and, if so, should the definition of idiopathic PD be expanded to include LB-negative disease?

Efforts to address some of these questions are now aided by the development of in vivo assays of α-synuclein aggregates, including CSF α-synuclein seeding amplification assays (SAAs) and skin α-synuclein immunofluorescence (IF). While methodological refinements and further development of these assays are ongoing, current evidence supports their use as biomarkers or in vivo proxies for Lewy pathology in the brain. These biomarkers are especially valuable given the absence of established imaging markers for Lewy pathology, in contrast to the availability of amyloid-β PET imaging in AD. Unlike postmortem histopathology, which captures a static end-stage snapshot, CSF α-synuclein SAAs and skin α-synuclein IF may provide insight into dynamic disease processes in living individuals [53]. An increasing number of studies have applied SAAs or IF to examine for differences between genotypes (Table 4) [53–62]. Across several of the α-synuclein SAA studies, CSF was positive in the majority of individuals with idiopathic PD (83% to 100%), and there tended to be an even higher proportion of positive SAAs among those with *GBA1*-associated PD (87% to 100%). In contrast, *LRRK2*-associated PD had a lower positivity rate (only 38% to 78%). One study found that the rate was lower in female *LRRK2* variant carriers (55%) and dropped further to 35% among *LRRK2* variant carriers with normal olfaction [53]. These findings suggest that α-synuclein aggregation may indeed be absent in a subset of *LRRK2*-associated PD, rather than LB-negative cases simply reflecting an artifact of timing or detection.

**Table 4:** Summary of α-synuclein biomarker positivity in idiopathic, *LRRK2*-associated, and *GBA1*-associated Parkinson’s disease

Report	Assay	Biospecimen	Genotype	*N*	Positivity rate (%)
Orru et al. 2025	SAA	CSF	iPD	764	92.0
			*LRRK2* PD	166	66.0
			*GBA1* PD	76	92.0
Schumacher et al. 2025	SAA	CSF	iPD	332	95.0
			*LRRK2* PD	162	54.1
			*GBA1* PD	70	93.9
Siderowf et al. 2023	SAA	CSF	iPD	373	93.3
			*LRRK2* PD	123	67.5
			*GBA1* PD	49	95.9
Chahine et al. 2025	SAA	CSF	iPD	378	100*
			*LRRK2* PD	148	69.0
Brockmann et al. 2021	SAA	CSF	iPD	107	91.0
			*LRRK2* PD	9	78.0
			*GBA1* PD	99	86.9
Fraser et al. 2026	SAA	CSF	iPD	14	100
			*LRRK2* PD	13	38.0
			*GBA1* PD	14	100
Garrido et al. 2019	SAA	CSF	iPD	10	90.0
			*LRRK2* PD	15	40.0
Garrido et al. 2023	SAA	CSF	iPD	6	83.3
			*LRRK2* PD	3	66.7
Yuan et al. 2024	IF	Skin	iPD	100	72.0
			*LRRK2* PD	14	78.6
			*GBA1* PD	5	40.0
Doppler et al. 2018	IF	Skin	*GBA1* PD	10	60.0

CSF=Cerebrospinal Fluid, IF=Immunofluorescence, SAA=Seed Amplification Assay

*Only SAA+ were included

To challenge the notion that α-synuclein aggregation can be entirely absent in *LRRK2*-associated PD, newer detection methods are beginning to uncover α-synuclein assemblies in LB-negative brains that evade detection by conventional immunohistochemistry. Two recent studies applied proximity ligation assay (PLA), a highly sensitive antibody-based technique that generates a fluorescent signal only when two specific antibodies, each conjugated to a DNA probe, bind to target epitopes in close proximity (typically within 40 nm). This method enables the visualization and quantification of oligomers that are too small or conformationally distinct to be detected by traditional, non-conformation-specific antibodies. Using PLA, both studies reported abundant α-synuclein oligomers in *LRRK2*-associated PD cases lacking classical Lewy pathology. Jensen and colleagues identified diffuse particulate α-synuclein signals in six LB-negative *LRRK2* cases: one with both p.G2019S and p.N2081D variants, and the other five cases carrying p.P1542S, p.M1646T, and/or p.N2081D variants [63]. Similarly, Sekiya and colleagues detected PLA-positive α-synuclein oligomers in brains from 19 individuals with *LRRK2* p.R1441C, p.G2019S, or p.I2020T variants despite an absence of conventional Lewy pathology. Notably, they observed an inverse correlation between oligomer burden and mature LB load [64]. Together, these findings suggest a model in which oligomeric α-synuclein may represent a stable and potentially toxic intermediate that either lies on the pathway to LB formation or reflects an alternative, non-Lewy pathology route. However, caution is warranted in interpreting these results, as the pathological relevance of the detected species remains uncertain, particularly given that oligomer-positive neurons are surviving rather than degenerated cells. PLA may also capture physiological oligomeric forms of α-synuclein, such as tetramers, under normal conditions. Additionally, Jensen and colleagues examined cases with *LRRK2* p.P1542S, p.M1646T, or p.N2081D which are not well-established pathogenic variants, raising the possibility that these individuals may have had idiopathic PD rather than *LRRK2*-mediated disease [63].

The observation by Jensen and colleagues that some cases, potentially representative of idiopathic PD, lack Lewy pathology adds to the growing body of evidence suggesting that idiopathic PD without LBs may constitute a distinct pathological entity. Earlier reports from brain bank studies have described LB-negative PD in approximately 20% of clinically diagnosed cases [65–67]. However, these studies generally lacked genotyping, and LB-negative cases were attributed to diagnostic error by the clinician rather than considered a possible subtype of PD in the absence of Lewy pathology. More recently, findings from the Parkinson’s Progression Markers Initiative (PPMI) cohort provide further support for this possibility. In that cohort, 7% of individuals with idiopathic PD were CSF α-synuclein SAA-negative, suggesting that a subset of PD patients may not exhibit Lewy pathology. To date, the existence of LB-negative idiopathic PD has been difficult to confirm. Traditional neuropathological diagnostic criteria for PD require the presence of Lewy pathology, and most brain banks classify tissue according to dominant histopathological features rather than clinical diagnoses. This introduces an archival bias, likely resulting in the underrepresentation or misclassification of clinically diagnosed PD cases that are pathologically LB-negative. To adequately address whether idiopathic PD without Lewy pathology represents a distinct pathological entity, systematic re-evaluation of archived brain tissue is needed from individuals diagnosed with PD by expert clinicians and confirmed to lack known pathogenic variants. Such efforts could help determine whether a bona fide LB-negative form of idiopathic PD exists and thus may share pathobiological features with *LRRK2*-associated PD.

## *LRRK2*- and *GBA1*-associated Lewy body disease as neuropathological subtypes at opposite ends of a spectrum

It is now well established that *LRRK2* and *GBA1* variants – the most common genetic risk factors for PD – are associated with contrasting neuropathological profiles. A subset of individuals with *LRRK2*-associated PD can lack LB pathology, while *GBA1*-associated PD and DLB are marked by a high burden of α-synuclein pathology, often involving cortical regions. AD co-pathology is generally limited in both genetic forms. These observations suggest that *LRRK2*- and *GBA1*-associated PD represent neuropathological subtypes at opposite ends of a spectrum of α-synuclein pathology in genetic PD (Fig. 1**)**.

The spectrum observed in genetic forms of PD offers a useful framework for conceptualizing potential subtypes within idiopathic PD. This model is based on the hypothesis that similar neuropathological subtypes may also occur in idiopathic PD among individuals without known pathogenic variants. Indeed, these proposed neuropathological subtypes appear to be mirrored by distinct clinical profiles. For example, some individuals with idiopathic PD experience a relatively benign course characterized by tremor-predominant motor symptoms, fewer non-motor features such as RBD or dementia, and later onset with slower progression, resembling a typical clinical presentation of *LRRK2*-associated PD. In contrast, others experience a more aggressive clinical trajectory marked by akinetic-rigid motor features, early RBD, more prominent cognitive impairment, and earlier onset with faster progression, aligning with a common clinical phenotype of *GBA1*-associated PD or DLB.

Extending the framework proposed above, we suggest that subsets of individuals with idiopathic PD may have “*LRRK2*-like” or “*GBA1*-like” disease, defined by patterns of neuropathological and clinical features. Between these two extremes likely lies a broader group of individuals with variable α-synuclein burden and increasing contributions from co-pathologies, such AD, TDP-43, or vascular pathologies. This intermediate zone may reflect alternate or mixed pathogenic drivers, overlapping neurodegenerative processes, as well as varying combinations of clinical features.

Recognizing *LRRK2*-like and *GBA1*-like subtypes within idiopathic PD has important implications. Mechanistically, such stratification may reflect LRRK2 hyperactivity or GCase dysfunction even in the absence of known pathogenic gene variants. In the case of LRRK2, this dysfunction could potentially occur through exposure to environmental pesticides such as rotenone, which has been shown to increase wild-type LRRK2 kinase activity in experimental models [68]. In the case of *GBA1*, α-synuclein accumulation can impair lysosomal activity of wild-type GCase [69]. Clinically, it underscores the heterogeneity of PD and reinforces the need for personalized approaches to prognosis and symptom management.

This framework has direct implications for disease-modifying treatment strategies in PD, particularly with respect to aligning therapeutic targets, presumed mechanisms of action, and participant selection in clinical trial design. If a therapy targeting LRRK2 or GCase is hypothesized to exert its disease-modifying effects primarily by reducing α-synuclein aggregation or downstream α-synuclein-mediated toxicity, then enrichment for participants with probable α-synuclein pathology would be expected to be important. This consideration is relevant for approaches such as small-molecule chaperones for GCase (e.g., ambroxol) since modulation of α-synuclein accumulation is central to the proposed mechanism of action [70]. By contrast, if LRRK2 or GCase dysfunction is presumed to contribute to neurodegeneration through mechanisms that are independent of α-synuclein aggregation, the presence of α-synuclein pathology may not be required for trial inclusion. For example, clinical trials of LRRK2 inhibitors designed to directly reduce kinase activity might focus on LRRK2 pathway hyperactivity for participant selection rather than α-synuclein status (e.g., NCT06680830). Importantly, this mechanistic framing implies that precision targeting of LRRK2 kinase hyperactivity or enhancement of GCase function may be relevant not only for individuals with confirmed genetic variants, but also for subsets of people with idiopathic PD in whom these pathways are dysregulated. Ultimately, this approach moves the field closer to a precision medicine framework grounded in pathobiological mechanisms rather than genetic labels alone.

## Conclusions

The contrasting pathological and mechanistic features of *LRRK2*- and *GBA1*-associated PD illustrate the heterogeneity that defines the broader spectrum of LBD. By positioning these genetic forms at opposite ends of a neuropathological spectrum, we propose a conceptual model in which idiopathic PD may encompass *LRRK2*-like and *GBA1*-like subtypes, each with distinct clinical trajectories, biomarker profiles, and underlying pathobiology. This framework helps reconcile diverse clinical presentations and pathological findings within idiopathic PD, including the growing recognition of LB-negative disease. Ultimately, a precision medicine approach grounded in pathobiological mechanisms, rather than clinical criteria or genetic status alone, may enhance our ability to interpret biomarkers, stratify patients, and develop targeted therapies for the wider PD population.

## Supplementary Information

Below is the link to the electronic supplementary material.

Supplementary Material 1

## Data Availability

No datasets were generated or analysed during the current study.

## Abbreviations

AD: Alzheimer’s disease
CNS: Central nervous system
COR: C-terminal of ROC domain
CSF: Cerebrospinal fluid
DLB: Dementia with Lewy bodies
GCase: Glucocerebrosidase
GD: Gaucher disease
GWAS: Genome wide association studies
LB: Lewy body
LBD: Lewy body disease
LRRK2: Leucine-rich repeat kinase 2
MSA: Multiple system atrophy
NFT: Neurofibrillary tangle
PLA: Proximity ligation assay
PD: Parkinson’s disease
PDD: Parkinson’s disease dementia
PPMI: Parkinson’s Progression Markers Initiative
RBD: REM sleep behaviour disorder
ROC: Ras of complex proteins domain
SAA: Seeding amplification assay
SN: Substantia nigra
