# Supplementary material for: LRRK2 and GBA1 in Lewy body diseases: neuropathological subtypes at opposite ends of a spectrum?
Source: Mol Neurodegener. 2026 Apr 27;21:32. doi: 10.1186/s13024-026-00939-z (PMC13267418; doi:10.1186/s13024-026-00939-z)
Supplement: Supplementary file 1 — Supplementary Material 1 [file 13024_2026_939_MOESM1_ESM.docx]

**Supplementary Table 1. Allele frequencies and variant classification of *GBA1* variants in Walton et al. 2024.**

| **Variant** | **Amino acid** | **Minor allele frequency** | **N** | **Consequence** | **ClinVar Classification** |
| --- | --- | --- | --- | --- | --- |
| rs2230288 | p.E365K | 3.19% | 57 | Missense | Conflicting classifications of pathogenicity & risk factor |
| rs75548401 | p.T408M | 1.38% | 25 | Missense | Conflicting classifications of pathogenicity |
| rs140335079 | x7−18 bp | 1.49% | 22 | Non-coding substitution | Benign/Likely benign |
| rs76763715 | p.N409S | 0.69% | 13 | Missense | Pathogenic/Likely pathogenic & Risk factor |
| rs421016 | p.L483P | 0.48% | 9 | Missense | Pathogenic/Likely pathogenic & Risk factor |
| rs80356771 | p.R502C | 0.27% | 5 | Missense | Pathogenic |
| rs41264927 | x1−15 bp | 0.21% | 4 | Non-coding substitution | Likely benign |
| rs368060 | p.A495P | 0.21% | 4 | Missense | Conflicting classifications of pathogenicity |
| rs1135675 | p.V499V | 0.21% | 4 | Synonymous | Benign/Likely benign |
| rs80356768 | p.L422PfsX3 | 0.16% | 3 | Frameshift | Absent |
| rs150466109 | p.K13R/p.K13T | 0.11% | 2 | Missense | Benign/Absent |
| rs147411159 | p.I158I | 0.21% | 2 | Synonymous | Conflicting classifications of pathogenicity |
| rs147138516 | p.D179H | 0.11% | 2 | Missense | Uncertain significance |
| rs367968666 | p.H294Q | 0.11% | 2 | Missense | Conflicting classifications of pathogenicity |
| rs140955685 | p.R301H | 0.11% | 2 | Missense | Uncertain significance |
| rs1064651 | p.D448H | 0.11% | 2 | Missense | Pathogenic/Likely pathogenic |
| rs368832292 | x11−12 bp | 0.11% | 2 | Non-coding substitution | Conflicting classifications of pathogenicity |
| rs104886460 | x2 + 1 bp | 0.05% | 1 | Splice site | Pathogenic/Likely pathogenic |
| Not applicable | x3−8 bp | 0.05% | 1 | Non-coding substitution | Absent |
| rs1141812 | p.R83C | 0.05% | 1 | Missense | Uncertain significance |
| Not applicable | x3 + 5 bp | 0.05% | 1 | Splice site | Absent |
| Not applicable | p.M124V | 0.05% | 1 | Missense | Uncertain significance |
| Not applicable | p.S146IfsX5 | 0.05% | 1 | Frameshift | Absent |
| rs439898 | p.R159W | 0.05% | 1 | Missense | Absent |
| Not applicable | p.A163T | 0.05% | 1 | Missense | Absent |
| rs398123530 | p.R170C | 0.05% | 1 | Recombination | Pathogenic |
| Not applicable | p.Y174C | 0.05% | 1 | Missense | Conflicting classifications of pathogenicity |
| rs774539868 | p.G234G | 0.05% | 1 | Synonymous | Likely benign |
| rs398123534 | p.G241R | 0.05% | 1 | Missense | Pathogenic |
| Not applicable | p.F290Y | 0.05% | 1 | Missense | Absent |
| rs78973108 | p.R296Q | 0.05% | 1 | Missense | Pathogenic |
| Not applicable | p.F298L | 0.05% | 1 | Missense | Absent |
| rs199628072 | p.T306I | 0.05% | 1 | Missense | Uncertain significance |
| rs753890133 | p.L325L | 0.05% | 1 | Synonymous | Absent |
| rs111417507 | x9−22 bp | 0.05% | 1 | Non-coding substitution | Absent |
| rs377143075 | x9−3 bp | 0.05% | 1 | Non-coding substitution | Absent |
| rs121908311 | p.G416S | 0.05% | 1 | Missense | Pathogenic/Likely pathogenic |
| Not applicable | p.R535C | 0.05% | 1 | Missense | Pathogenic |

Pathogenicity and risk factor status are reported according to current ClinVar entries, where available (adapted from Walton et al. 2024).
